# A Rare Case of Sterile Retrostyloid Abscess in a Patient With Crohn’s Disease: Diagnostic and Therapeutic Insights

**DOI:** 10.1155/carm/8720602

**Published:** 2026-05-14

**Authors:** Giorgio Babino, Vito Sala, Barbara Verro, Antonio Lo Casto, Carmelo Saraniti

**Affiliations:** ^1^ Department of Biomedicine, Neuroscience and Advanced Diagnostic, Division of Otorhinolaryngology, University of Palermo, 90127, Palermo, Italy, unipa.it; ^2^ Department of Biomedicine, Neuroscience and Advanced Diagnostic, Division of Radiology, University of Palermo, 90127, Palermo, Italy, unipa.it

**Keywords:** abscess, Crohn disease, inflammatory bowel diseases, neck abscess, parapharyngeal space

## Abstract

**Introduction:**

Aseptic abscess syndrome is a rare inflammatory disorder of unclear etiology, characterized by sterile lesions densely infiltrated by polymorphonuclear neutrophils. Its frequently associated with inflammatory bowel disease, which includes chronic gastrointestinal disorders such as Crohn’s disease and ulcerative colitis, often accompanied by both intestinal and extraintestinal manifestations. Extraintestinal involvement may include musculoskeletal, cutaneous, ocular, oral, and hepatobiliary systems. Although uncommon, aseptic abscess syndrome can precede the onset of inflammatory bowel disease or occur during its course.

**Case Report:**

A 44‐year‐old woman with Crohn’s disease receiving corticosteroid therapy is presented with abdominal pain, diarrhea, rectal bleeding, and a painful right lateral cervical swelling with trismus. Contrast‐enhanced computed tomography revealed a right retrostyloid abscess. Surgical drainage, combined with antibiotics and corticosteroids, led to clinical resolution. Cultures were negative, confirming the diagnosis of aseptic abscess.

**Conclusion:**

Retrostyloid abscesses are extremely rare and poorly documented. In patients with inflammatory bowel disease presenting with deep neck abscesses, aseptic abscess syndrome should be considered, as early recognition and multidisciplinary management are essential to prevent serious complications.

**Key Messages**
• Sterile deep neck abscesses should be considered in IBD patients with negative cultures.
• Retrostyloid abscesses are a rare manifestation of aseptic abscess syndrome.
• Early immunosuppressive treatment is crucial for resolution and complication prevention.

## 1. Introduction

Deep neck abscesses are potentially life‐threatening emergencies, most commonly arising from odontogenic or upper airway infections. Among these, retrostyloid abscesses are extremely rare, involving the retrostyloid space, a deep anatomical region adjacent to critical neurovascular structures, including the carotid artery, internal jugular vein, vagus, glossopharyngeal, accessory, and hypoglossal nerves, the sympathetic chain, and lymphatics.

Clinical presentation is often nonspecific and may include cervical pain and swelling, dysphagia, odynophagia, trismus, fever, and stomatolalia, which can delay diagnosis. Contrast‐enhanced computed tomography (CT) is crucial for accurate localization and assessment of abscess extent. Delayed treatment may result in severe complications, including vascular (e.g., jugular vein thrombosis, venous septic embolus, carotid artery pseudoaneurysm or rupture, disseminated intravascular coagulopathy), neurological (e.g., Horner’s syndrome, cranial‐nerve IX, X, XI, and XII deficits), thoracic (e.g., mediastinitis, pericarditis, and pleural empyema), and intracranial (e.g., meningitis and cerebral abscesses) events [1].

Sterile abscess syndrome, as inflammatory neutrophil‐mediated disorder, first described by André et al., is frequently associated with inflammatory bowel disease (IBD). It is characterized by persistent fever (up to 90% of cases), elevated leukocyte counts, sterile blood cultures, and formation of sterile abscesses at various anatomical sites [2, 3]. Abdominal involvement, particularly the spleen, is most common, although abscesses may develop elsewhere. In patients with IBD, AAS has been reported in up to 70% of cases [4, 5].

Herein, we describe a case of a sterile retrostyloid abscess in a patient with Crohn’s disease undergoing immunosuppressive therapy, highlighting diagnostic and therapeutic challenges and contextualizing it within the limited existing literature.

## 2. Case Presentation

A 44‐year‐old woman is presented to our ENT clinic with a 1‐week history of painful right submandibular swelling and trismus, accompanied by abdominal pain, diarrhea, and rectal bleeding, suggestive of IBD exacerbation. She had a history of Crohn’s disease complicated by pyoderma gangrenosum and uveitis. Previous treatments included infliximab and adalimumab, discontinued due to adverse reactions or secondary loss of efficacy. Since 2020, she had not attended regular gastroenterology follow‐up and had self‐initiated prednisone therapy (25 mg/day) two months prior due to symptom flare‐up.

Laboratory evaluation revealed elevated C‐reactive protein (CRP) (115 mg/L), severe anemia (Hb 5.9 g/dL), and normal white blood cell count (10.45 × 10^3^/μL). Flexible fiberoptic endoscopy was performed as part of the routine evaluation of deep neck infections to assess airway patency and exclude mucosal or neoplastic lesions of the upper aerodigestive tract. The examination demonstrated medialization of the right pharyngeal wall at the oropharyngeal and nasopharyngeal levels, without involvement of the hypopharynx or larynx. However, given the emergency clinical presentation and the need for immediate surgical intervention, laryngoscopic images were not recorded during the initial evaluation.

Contrast‐enhanced CT of the neck revealed a right lateral cervical abscess (38 × 16 mm) originating from the tonsillar fossa, with posterior extension involving the levator scapulae muscle and likely the longus colli muscle (Figure 1).

FIGURE 1Contrast‐enhanced axial computed tomography (CT) scan of the neck demonstrating a well‐defined hypodense fluid collection (38 × 16 mm) in the right retrostyloid compartment of the parapharyngeal space (red arrow). The lesion shows peripheral rim enhancement after contrast administration, consistent with abscess formation. The collection displaces the carotid sheath structures anteromedially and extends posteriorly toward the prevertebral musculature, involving the levator scapulae and likely the longus colli muscle. No vascular thrombosis or airway compression is evident.

**Figure:**
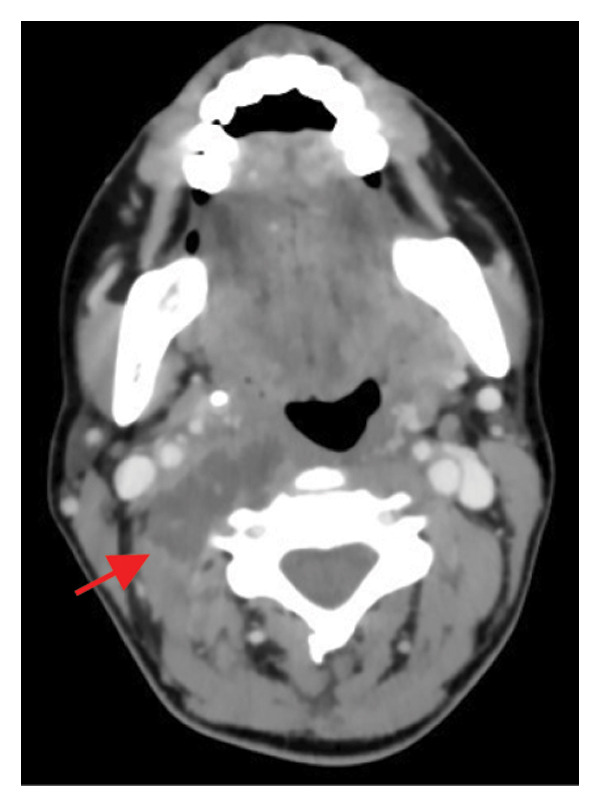
(a)

**Figure:**
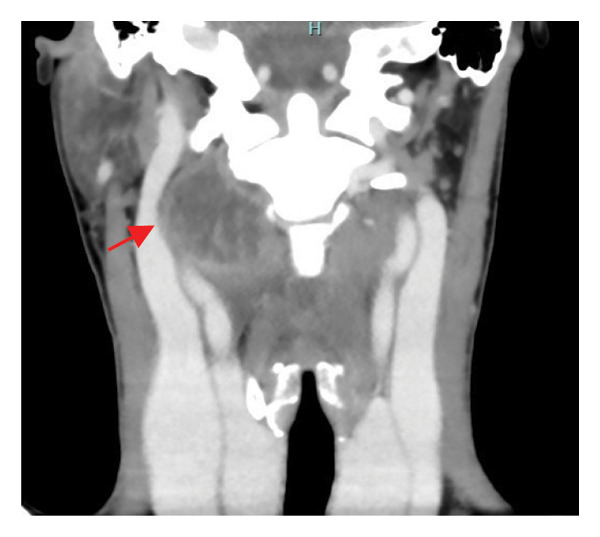
(b)

**Figure:**
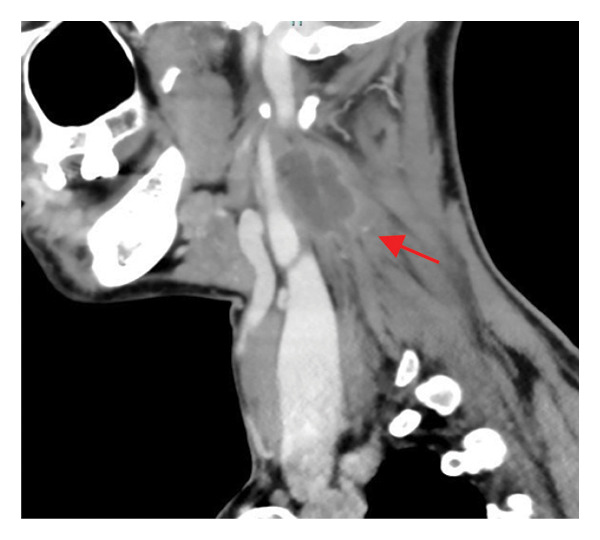
(c)

**FIGURE 2:**
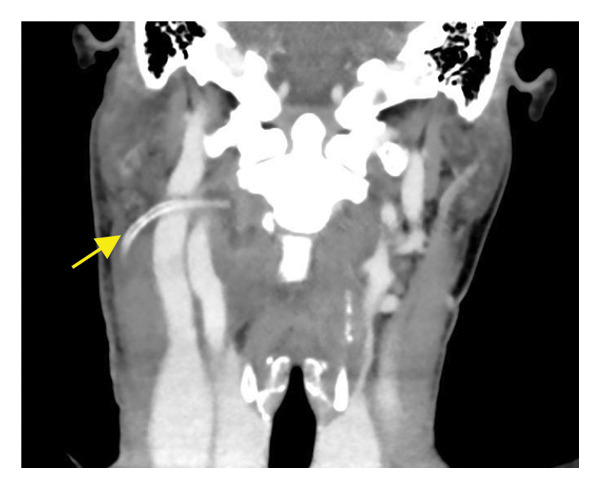
Postoperative contrast‐enhanced CT scan (coronal view) showing resolution of the previously described fluid collection and the drainage catheter in situ.

The patient underwent cervicotomy drainage, combined with broad‐spectrum intravenous antibiotics (piperacillin/tazobactam 4.5 g, administered four times daily until the fifth postoperative day) and high‐dose corticosteroids (methylprednisolone 1 g daily), as recommended by the infectious disease specialist. She also received blood transfusions for anemia management. Microbiological cultures were negative, confirming a sterile abscess. Postoperative contrast‐enhanced CT performed on Day 4 demonstrated complete resolution of the previously described fluid collection, with no residual hypodense area, no peripheral rim enhancement, and restoration of normal parapharyngeal fat planes (Figure 2).

There was no evidence of vascular thrombosis, airway compromise, or mediastinal extension. Clinically, resolution was defined by disappearance of cervical swelling and trismus, absence of fever, progressive normalization of inflammatory markers, and improvement of abdominal symptoms following corticosteroid therapy. Therefore, antibiotics were discontinued, and the patient was subsequently managed for residual gastrointestinal symptoms. Corticosteroid therapy (methylprednisolone) was gradually tapered from 1 g/day intravenously to 250 mg/day intravenously. Beginning on postoperative Day 5, upon the gastroenterologist’s recommendation, the dosage was further reduced to 60 mg/day intravenously for 10 days. Therapy was subsequently transitioned to oral prednisone 50 mg once daily (morning administration), which was continued until complete clinical remission was achieved (for an additional 5 days). Thereafter, the patient was discharged with instructions to continue oral prednisone 37.5 mg once daily (morning administration) until the scheduled gastroenterology follow‐up. She was discharged on Day 20 with full recovery, and follow‐up showed no recurrence.

## 3. Discussion

Parapharyngeal abscesses, including retrostyloid variants, are rare, with an estimated incidence of 0.9–2.6 per 100,000, and up to 52% are associated with concomitant peritonsillar abscess [6]. A recent study investigating the epidemiological characteristics of parapharyngeal and retropharyngeal abscesses (PRPA) in the general adult population of Taiwan estimated an annual incidence of approximately 2.64 per 100,000 people, with a slight male predominance (M:F ratio = 1.72:1) and a higher prevalence in the 45–64 and 64+ age groups, and a lower prevalence in the 20–44 age group [7].

On the contrary, our patient, a 44‐year‐old female, represents an uncommon demographic.

Systemic immunosuppression, including that induced by IBD therapy, increases susceptibility. Diabetes is the most commonly associated systemic disease, but other immunosuppressive states, such as corticosteroid therapy, may also predispose patients to abscess formation [8]. In our case, the patient was in a state of iatrogenic immunosuppression related to the medical treatment of Crohn’s disease [9].

Common etiologies include odontogenic and upper respiratory infections. Anaerobes (73%) and Streptococcus species (67%) are commonly isolated, but cultures may be negative in sterile abscess syndrome, as in this case. Contrast‐enhanced CT remains the gold standard for diagnosis, with reported sensitivity of 100% [10]. Magnetic resonance imaging (MRI) provides superior soft‐tissue resolution but is usually reserved for selected cases [11]. In suspected odontogenic origin, orthopantomography may provide additional information [12]. Surgical drainage, via transcervical or transoral approach, remains the mainstay, complemented by antibiotics [2, 13, 14]. In our patient, cervicotomy was performed, providing access posterior to the vascular‐nervous axis.

AAS is characterized by polymorphonuclear neutrophils‐rich sterile lesions, often associated with IBD [2]. The syndrome may occur concurrently with IBD, after a variable interval following diagnosis, or, occasionally, as the initial manifestation of IBD. This highlights the potential need for diagnostic procedures such as gastroscopy or colonoscopy in otherwise asymptomatic patients to enable early detection [2, 15, 16]. Commonly affected sites include the spleen, lymph nodes, skin, liver, and lungs, though other locations may also be involved.

André et al. reported a 54‐year‐old woman with ulcerative colitis who developed an aseptic retropharyngeal abscess, successfully treated with intravenous methylprednisolone and cyclophosphamide [3]. Debourdeau et al. described an 18‐year‐old male patient with Crohn’s disease on infliximab who presented with laterocervical lymphadenopathy; histological analysis revealed noncaseating epithelioid granulomas. The collection did not respond to initial antituberculous therapy but resolved completely following intravenous corticosteroids [14]. Trefond et al. conducted a French study of 71 patients meeting the criteria for sterile abscesses outlined by André et al. [5]. Among these, 37 patients (52%) were men, with a mean age of 34.5 ± 17 years. Abscesses were located in the spleen (71.8%), lymph nodes (50.7%), skin (29.5%), liver (28.1%), and lungs (22.5%). Only 5 cases (7%) involved the ENT region, though the precise sites were not clearly specified. Corticosteroids and/or immunosuppressive agents constituted the primary treatment, and 42% of patients had concurrent IBD [2].

Although sterile abscesses associated with IBD have been described, to our knowledge, this is the first documented case of a sterile retrostyloid abscess associated with IBD [2, 3, 14] (Table 1).

**TABLE 1:** Neck abscesses associated with inflammatory bowel disease.

Study	Sex/age	Abscess site	Comorbidity	Laboratory findings	Microbiological cultures	Treatment	Outcome
Doulberis et al. [17]	F, 36	Retropharyngeal abscess	Peripheral spondyloarthritis and Crohn disease treated with CS	Elevated CRP levels	Streptococcus constellatus	Antibiotic therapy and surgical drainage	Complete resolution
André et al. [3]	F, 54	Retropharyngeal abscess	Pyoderma gangrenosum, UC treated with 5‐ASA, steroids during flares	Elevated Erythrocyte sedimentation rate, CRP and leukocyte count levels	Sterile	Fluid aspiration, CS and antibiotic therapy	1 month: painful retropharyngeal and laterocervical muscular collections + subcutaneous cervical and splenic abscesses; microbiology negative; treated with steroids + cyclophosphamide. 3 years: UC relapse with pyoderma gangrenosum and isolated splenic abscesses; treated with steroids + cyclophosphamide.
Debourdeau et al. [14]	M, 18	Laterocervical lymphadenopathy	Crohn disease treated with infliximab	Elevated CRP levels	Sterile	CS therapy	Complete resolution
Nuriyev [18]	F, 31	Retropharyngeal microabscess	Crohn disease treated with vedolizumab and azathioprine voluntarily discontinued after the patient became pregnant	Elevated CRP levels	Not performed	Antibiotic therapy	Complete resolution
Our study	F, 44	Retrostyloid abscess	Crohn disease under treatment with prednisone	Elevated CRP levels and normal white blood cell count	Sterile	Surgical drainage, antibiotic and steroid therapy	Complete resolution

*Note:* yrs: years.

Abbreviations: CRP, C‐reactive protein; CS, corticosteroids; UC, ulcerative colitis.

Biochemical analyses typically reveal elevated CRP and leukocytosis; in our case, CRP was elevated, but leukocyte counts remained normal, likely due to steroid‐induced myelosuppression. The combination of persistent abdominal symptoms, elevated CRP, and negative cultures supports an inflammatory rather than infectious etiology.

Management of sterile abscesses primarily involves corticosteroids or immunosuppressive therapy, with surgical drainage reserved for cases requiring immediate decompression or diagnostic confirmation [1, 14]. In our case, abscess resolution was established using combined clinical and radiological criteria. Radiologically, complete resolution was defined as the disappearance of the fluid collection and peripheral contrast enhancement on CT imaging, with re‐expansion of adjacent anatomical compartments. Clinically, the patient exhibited full regression of local inflammatory signs and rapid response to corticosteroid therapy. The absence of persistent collection despite early discontinuation of antibiotics further supported the inflammatory rather than infectious nature of the lesion.

A limitation of this report is the absence of histopathological examination of the abscess wall. Surgical drainage was performed for therapeutic purposes in an emergency context, and no tissue biopsy was obtained. Therefore, the diagnosis of aseptic abscess was based on clinical presentation, negative microbiological cultures, association with active Crohn’s disease, and favorable response to corticosteroid therapy. Although histology typically demonstrates dense neutrophilic infiltrates without microbial evidence, this was not available in our case.

## 4. Conclusion

Although rare, parapharyngeal abscesses can be life‐threatening, and their nonspecific presentation may delay diagnosis. This case highlights the critical importance of timely recognition and intervention, which in our patient prevented serious complications and ensured complete recovery.

Optimal management requires a multidisciplinary approach, involving radiologists, infectious disease specialists, thoracic surgeons, ENT specialists, and anesthesiologists. Accurate interpretation of clinical and radiological findings is essential to determine abscess type, select the most appropriate surgical approach, and optimize treatment timing.

Importantly, this report underscores the under‐recognized but clinically significant association between IBD and AAS. Clinicians should consider this diagnosis in patients with IBD presenting with deep neck abscesses, as early immunosuppressive therapy can improve outcomes, avoid unnecessary antibiotics, and expand understanding of AAS presentations in immunocompromised patients.

This case not only expands the spectrum of known presentations of aseptic abscess syndrome but also serves as a reminder of the need for vigilance in immunocompromised patients with atypical abscesses.

## Funding

No funding was received for this manuscript.

Open access publishing facilitated by Universita degli Studi di Palermo, as part of the Wiley ‐ CRUI‐CARE agreement.

## Ethics Statement

Written informed consent was obtained from the patient for publication of this case report.

## Conflicts of Interest

The authors declare no conflicts of interest.

## Data Availability

The data that support the findings of this study are available in PubMed at https://pubmed.ncbi.nlm.nih.gov. These data were derived from the following resources available in the public domain: abscess sterile crohn, https://pubmed.ncbi.nlm.nih.gov/?term=abscess+sterile+crohn.
